# Splanchnic Haemodynamics and Vasoactive Agents in Experimental Cirrhosis

**DOI:** 10.1155/1994/52975

**Published:** 1994

**Authors:** J. G. Geraghty, W. J. Angerson, D. C. Carter

**Affiliations:** University Department of Surgery Royal Infirmary Glasgow UK

## Abstract

It is well known that portal hypertension is associated with a hyperdynamic systemic circulatory state. This study measures systemic and splanchnic haemodynamics in an experimental rat model of hepatic cirrhosis. It also investigates the association between haemodynamic changes in cirrhotic animals and circulating levels of the vasoactive hormones glucagon and vasoactive intestinal polypeptide (VIP). Splanchnic blood flow was significantly increased in the cirrhotic group (13.2 ± 1.3 vs. 9.2 ± 1.6 ml/min, *P* < 0.05). Circulating levels of glucagon and VIP were two and five fold increased respectively in cirrhotic animals compared to controls. There was a strong correlation between portal pressure and glucagon levels in the cirrhotic group (*r* = 0.85). Raised splanchnic blood flow is partly responsible for elevated portal pressure in this model and this rise may be humorally mediated.


*HPB Surgery*, 1994, Vol. 8, pp. 83–88
Reprints available directly from the publisher
Photocopying permitted by license only

© 1994 Harwood Academic Publishers GmbH
Printed in Malaysia

### Splanchnic Haemodynamics and Vasoactive Agents in Experimental Cirrhosis

J. G. GERAGHTY, W. J. ANGERSON and D. C. CARTER

University Department of Surgery, Royal Infirmary, Glasgow, U.K.

It is well known that portal hypertension is associated with a hyperdynamic systemic circulatory state. This study measures systemic and splanchnic haemodynamics in an experimental rat model of hepatic cirrhosis. It also investigates the association between haemodynamic changes in cirrhotic animals and circulating levels of the vasoactive hormones glucagon and vasoactive intestinal polypeptide (VIP). Splanchnic blood flow was significantly increased in the cirrhotic group ($13.2 \pm 1.3$ vs. $9.2 \pm 1.6$ ml/min, $P < 0.05$). Circulating levels of glucagon and VIP were two and five fold increased respectively in cirrhotic animals compared to controls. There was a strong correlation between portal pressure and glucagon levels in the cirrhotic group ($r = 0.85$). Raised splanchnic blood flow is partly responsible for elevated portal pressure in this model and this rise may be humorally mediated.

### INTRODUCTION

It has been well established that portal hypertension is associated with hyperdynamic systemic and splanchnic circulatory states[1,2]. It has also been demonstrated that raised splanchnic blood flow plays an important role in the maintenance of a chronically elevated portal pressure in experimental portal hypertension[3,4]. Several theories have been proposed to explain the splanchnic hyperaemia observed in portal hypertension including decreased sensitivity of splanchnic arterioles to noradrenaline[5] and the opening of intestinal arteriovenous shunts[6]. It has also been suggested that circulating vasoactive agents, normally catabolised by the liver, are present in increased quantities in hepatic cirrhosis and may mediate these haemodynamic changes[7]. Glucagon, a potent intestinal vasodilator, is present in increased concentration in the plasma of patients and animals with portal hypertension and has been implicated in the intestinal hyperaemia associated with this condition[8–10]. The present study defines the systemic and splanchnic haemodynamics changes in an animal model of experimental cirrhosis, and assesses their relationships with circulating concentration of putative splanchnic vasodilators (glucagon and vasoactive intestinal peptide).

### MATERIALS AND METHODS

Male Sprague-Dawley rats (Bantin and Kingman Ltd. UK) were housed in a controlled environment with a 12-hour light: dark cycle. They were fed standard rat diet but were fasted overnight in wire bottom cages prior to measurement of splanchnic haemodynamics.

### Production of Experimental Cirrhosis

Hepatic cirrhosis was produced using oral administration of carbon tetrachloride ($CCl_4$) as previously described[11]. Animals weighing $150 \pm 20$ gm were given phenobarbitol (150/100 ml) in their drinking water to induce the enzyme cytochrome P450 which has been shown to increase rat liver sensitivity to $CCl_4$[12]. This hepatotoxin was given once weekly using an orogastric tube under light ether anaesthesia. The initial dose was 0.15 ml and each subsequent weekly dose was calculated according to the animals weight loss following the previous dose. Carbon tetrachloride was stopped as soon as ascites developed and experiments were performed 3 weeks later. Control animals of similar weight also received phenobarbitol by weekly gavage under ether anaesthesia and were weighed daily; however they did not receive carbon tetrachloride. We have previously found[11] that 40% of animals receiving $CCl_4$ as described above will develop ascites and micronodular hepatic cirrhosis (Figure 1). A total of 15 animals were gavaged with $CCl_4$ and the six animals which developed ascites in this group had haemodynamic experiments performed.

### Surgical Preparation

Systemic and splanchnic haemodynamics were measured using a reference microsphere sample technique[13]. Each animal had a tracheostomy performed and was ventilated using a Harvard rat ventilator (model 68) with a 2:1 nitrous oxide—oxygen mixture in 0.5% halothane. The femoral artery was cannulated on both sides and mean arterial pressure recorded using a strain gauge transducer connected to a Gould 8000 semi-dual recorder. Arterial blood gas concentrations were analysed (ABL, Radiometer) and only animals with a P02 over 90 mmHg, a PC02 in the range 35–42 mmHg and a pH greater than 7.35 were included in the study. Rectal temperature was monitored and kept at $37 \pm 0.5$ C by means of a heat lamp. The abdomen was opened using a midline incision and a cannula inserted into the ileocolic branch of the portal vein to measure portal venous pressure. The abdomen was closed and portal pressure measured a minimum of 30 minutes later and only when a respiratory pattern was present on portal venous tracing. Mean arterial and portal pressure measurements were taken simultaneously. Finally a cannula was inserted into the left ventricle via the right carotid artery for injection of radio-labelled microspheres. Insertion was performed under continuous pressure monitoring and correct positioning was verified both by the presence of a ventricular pattern on pressure recording and by checking the position of the tip of the cannula at the end of the experiment.

### Haemodynamic Measurements

Once the animal was haemodynamically stable and pressure measurements had been recorded, approximately 100,000 Ruthenium-labelled microspheres were injected into the left ventricle over 25 seconds[13]. The microspheres were suspended in 10% Dextran with a drop of 0.01% "Tween" 80 added to prevent clumping. The amount of radioactivity in the injection syringe (volume = 0.2 ml) was measured in a gamma-scintillation counter (Packard 5000) and the syringe was vortexed for 30 seconds prior to injection. Arterial pressure was recorded continuously during infusion and a change in pressure of more than 10% over basal value led to automatic exclusion.

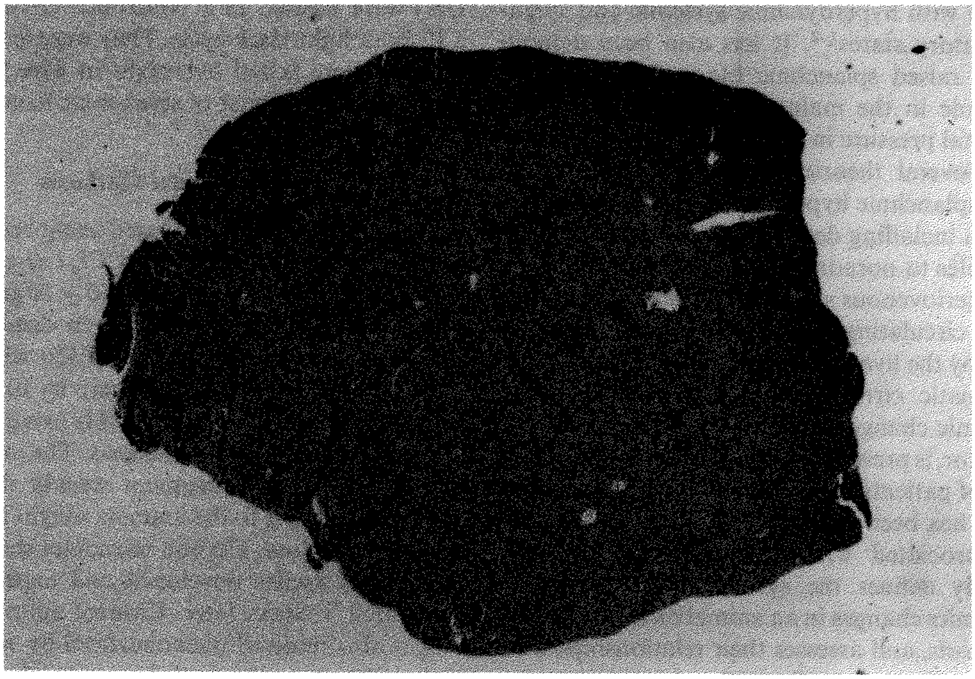

**Figure 1** Histological section of liver taken from an animal with experimental cirrhosis.

The femoral artery cannula was connected to a peripheral 2 ml syringe, which was attached to a withdrawal pump (Sage Instruments). Withdrawal was commenced 10 seconds before intra-ventricular injection of microspheres and stopped 60 seconds later. The reference sample in the syringe therefore served as an "artificial organ" with a known blood flow (1 ml/min). The radioactivity in the syringe was counted in a gamma-scintillation counter. The degree of portasystemic shunting was measured by injecting 100,000 Tn-labelled microspheres into the ileocolic branch of the portal vein as previously described[14]. The animal was killed 2 minutes later and the lungs, liver, splanchnic organs and kidneys were removed and placed in vials for measurement of radioactivity.

### Calculations

Cardiac output (ml/min)

$$= \frac{\text{Injected radioactivity (dpm)} \times \text{Reference organ flow (ml/min)}}{\text{Reference organ radioactivity (dpm)}}$$

Organ blood flow (ml/min)

$$= \frac{\text{Organ radioactivity (dpm)} \times \text{Reference organ flow (ml/min)}}{\text{Reference organ radioactivity (dpm)}}$$

Portal venous inflow was the sum of flows to the stomach, spleen, small and large bowel, and mesentery.

Vascular resistance (mm Hg/ml/min)

$$= \frac{\text{Change in pressure across vascular bed (mm Hg)}}{\text{Blood flow to vascular bed (ml/min)}}$$

Portasystemic Shunting (%)

$$= \frac{\text{Lung radioactivity (dpm)}}{\text{Liver + lung radioactivity (dpm)}} \times 100$$

Uniform mixing of microspheres in blood was checked by comparing radioactivity in both kidneys and animals with a greater than 10% difference were excluded.

### Measurement of Glucagon and VIP

At the end of the experiment blood was taken from the inferior vena cava for measurement of glucagon and vasoactive intestinal polypeptide levels. Each sample was placed in a heparinised tube and frozen. A radioimmunoassay technique was used to measure glucagon and vasoactive intestinal polypeptide[15,16].

### Statistical Analysis

Results are presented as mean ± standard error of the mean (mean ± SEM).The statistical tests used are the Mann-Whitney and Pearsons correlation coefficient.

### RESULTS

### Systemic and splanchnic haemodynamics

Portal venous pressure was significantly higher in cirrhotic animals compared to controls (Table 1). Portasystemic shunting was not detectable in controls but 23.4% portal blood was diverted into the systemic circulation in cirrhotic animals.

Portal hypertension was associated with a rise in cardiac output and reduced total portal resistance (Table 1). There was a significant rise in splanchnic blood flow which was associated with a reduction in splanchnic arteriolar resistance. Total portal resistance was 50% greater in cirrhotic animals but this difference compared to controls was not statistically significant.

### Hormonal Concentrations

There was a five fold increase in the level of vasoactive intestinal polypeptide in the cirrhotic group compared to controls (Table 2). However there was no correlation between portal pressure and levels of vasoactive intestinal polypeptide in cirrhotic animals. Although glucagon levels were not significantly increased in cirrhotic animals (Table 2) there was a strong correlation ($r = 0.85$) between portal venous pressure and glucagon levels (Figure 2). There was no correlation between glucagon levels and the magnitude of portasystemic shunting.

**Table 1** Haemodynamic comparison between cirrhotic and control groups.

| | *Cirrhosis* ($n = 6$) | *Control* ($n = 6$) |
|---|---|---|
| Portal pressure (mmHg) | 15.8 ± 1.4 | 6.4 ± 0.3 |
| Portasystemic shunting (%) | 23.4 ± 3.2 | 0 |
| Cardiac output (ml/min) | 172.5 ± 45.4 | 116.7 ± 20.9 |
| Total peripheral (mmHg/ml/min) resistance | 1.1 ± 0.1 | 1.7 ± 0.2* |
| Splanchnic inflow (ml/min) | 13.2 ± 1.3 | 9.2 ± 1.6* |
| Splanchnic arteriolar (mmHg/ml/min) resistance | 8.0 ± 1.3 | 15.1 ± 3.1* |
| Total portal resistance (mmHg/ml/min) | 1.2 ± 0.1 | 0.8 ± 0.1* |

Values are mean ± SEM; * $p < 0.01$

**Table 2** Hormonal levels in cirrhotic and control groups.

| | *Cirrhosis* ($n = 6$) | *Control* ($n = 6$) | *p-value* |
|---|---|---|---|
| Glucagon (ng/L) | 865 ± 135 | 481 ± 152 | 0.09 |
| Vasoactive intestinal polypeptide (ng/L) | 150 ± 32 | 31 ± 17 | 0.01 |

Values are mean ± SEM

### DISCUSSION

This study shows that experimental cirrhosis is associated with hyperdynamic systemic and splanchnic circulatory states. These results are in agreement with previous observations that raised splanchnic inflow is the primary factor in the maintenance of an elevated portal pressure in experimental portal hypertension[1,2,3]. It has been suggested that this rise in splanchnic inflow may be hormonally mediated[7]. Glucagon and vasoactive intestinal polypeptide are known to increase splanchnic blood flow. These hormones are produced by splanchnic organs and are normally catabolised by the liver. In portal hypertension however, glucagon and vasoactive intestinal polypeptide may escape into the systemic circulation via portasystemic collateral vessels.

The second aim of the present study therefore was to measure the levels of glucagon and vasoactive intestinal peptide in portal hypertension and to establish whether they were associated with the rise in portal pressure seen in experimental cirrhosis. The results show that both glucagon and vasoactive intestinal polypeptide levels are increased in experimental cirrhosis. They also demonstrate a strong correlation between portal pressure and glucagon levels suggesting that this agent may be responsible in part for the rise in portal pressure seen in cirrhotic animals. In contrast there was no association between circulating levels of vasoactive intestinal polypeptide and portal venous pressure. It is not clear why these hormones are raised in the model of cirrhosis. One possible mechanism is that they pass directly into the systemic circulation in portasystemic collateral vessels and therefore escape catabolism in the liver. There was however no correlation between circulating levels of glucagon and the magnitude of portasystemic shunting seen in animals with cirrhosis. Portasystemic collateral vessels are only partly responsible however for physiological shunting in portal hypertension. Reduced catabolism of substances normally degraded by the liver will also give rise to physiological shunting in hepatic cirrhosis. It is possible that the rise in glucagon levels in portal hypertensive animals in the present study may be due in part to reduced catabolism by a cirrhotic liver. Pancreatic hypersecretion of glucagon is known to occur in experimental portal hypertension and this could have also contributed to the raised glucagon levels observed in the present study[17].

There is now strong experimental evidence to suggest that humoral factors mediate the splanchnic hyperaemia seen in chronic prehepatic portal hypertension. It has been shown that cross-perfusion of the mesenteric circulation of normal rats with arterial blood from rats with portal hypertension results in a 30% increase in intestinal blood flow[10].

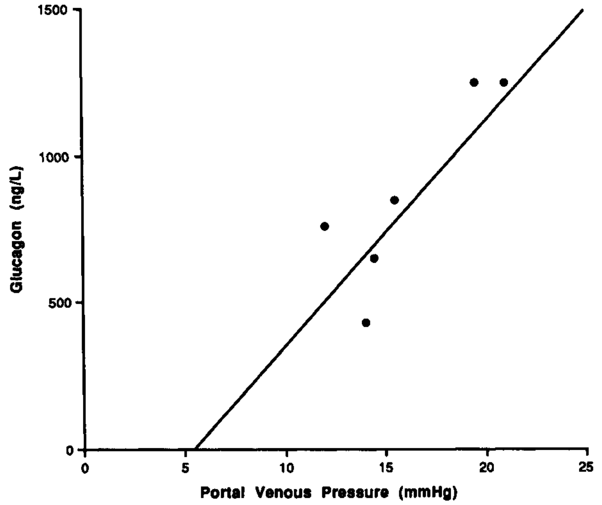

**Figure 2** There was a strong correlation between glucagon levels and portal pressure measurements in cirrhotic animals.

Recent studies have concentrated on the role of glucagon as a possible mediator of this response as it reduces intestinal arteriolar resistance and levels are known to be elevated in the plasma of patients and animals with portal hypertension[8,9]. This hypothesis was tested by Benoit and colleagues[10] who showed that administration of glucagon antiserum caused a 30% reduction in portal venous inflow in rats with portal hypertension.

The present study differs from previous studies in that it measures splanchnic haemodynamics, glucagon and vasoactive intestinal polypeptide levels, and portal venous pressure in experimental cirrhosis. The results support the contention of Benoit *et al.* that glucagon plays a role in the maintenance of a raised portal pressure in portal hypertension[7,10]. Given that resistance to portal blood flow also plays a role in elevating portal pressure, it is likely that there is a synergistic effect between increased portal venous inflow and resistance to flow resulting in portal hypertension. Raised portal pressure in hepatic cirrhosis could therefore be explained by the following sequence of events. As cirrhosis develops, portal pressure rises and beyond a critical pressure portasystemic collateral vessels open. Humoral agents such as glucagon are released into the systemic circulation through these shunts giving rise to a reduction in splanchnic arteriolar resistance. The resulting increase in splanchnic blood flow then produces a rise in portal venous pressure. The evidence supporting this hypothesis however is limited almost exclusively to animal studies. Similar experiments in humans are difficult to perform because of the lack of established non-invasive techniques to measure splanchnic blood flow, magnitude of portasystemic shunting and portal venous pressure. The present study suggests that glucagon may play a role in the maintenance of raised portal pressure but this hypothesis needs to be tested in a clinical setting.

### ACKNOWLEDGEMENTS

This work was supported by a grant from the Scottish Hospitals Endowment Research Trust.

### REFERENCES

1. Vorobioff, J., Bredfeldt, J. E. and Groszmann, R. J., (1983) Hyperdynamic circulation in portal-hypertensive rat model: a primary factor for maintenance of chronic portal hypertension. *Am. J. Physiol.*, **244**, G52–57.
2. Murray, J. F., Dawson, A. M. and Sherlock, S., (1958) Circulatory changes in chronic liver disease *Am. J. Med.* **24**, 358–367.
3. Sikular, E., Kravetz, E. and Groszmann, R. J., (1985) Evolution of portal hypertension and mechanisms involved in its maintenance in a rat model. *Am. J. Physiol.*, **248**, G618–625.
4. Benoit, J. N., Womack, W. A., Hernandez, L. and Granger, D. N., (1985) "Forward" and "Backward" flow mechanisms of portal hypertension. *Gastroenterology.*, **89**, 1092–1096.
5. Keil, J. W., Pitts, V., Benoit, J. N., Granger D. N. and Shepherd, A. P. (1985) Reduced vascular sensitivity to norepinephrine in portal-hypertensive rats. *Am. J. Physiol.*, **248**, G192–195.
6. Hashizume, M., Tanaka M. and Inokuchi, L. Morphology of the gastric microcirculation in cirrhosis. *Hepatology*, **3**, 1008–1012.
7. Benoit, J. N., Barrowman, J. A., Harper, S. L., Kvietys P. R. and Granger, D. N. (1984) Role of humoral factors in the intestinal hyperaemia associated with chronic portal hypertension. *Am. J. Physiol.*, **247**, G486–493.
8. Macro, J., Diego, J., Villannova, M. L., Diaz-Fierros, M., Valverde I. and Segovia, J. M. (1973) Elevated plasma glucagon levels in cirrhosis of the liver, *N. Engl. J. Med.*, **289**, 1107–1111.
9. Sherwin, R., Joshi, P., Handler, R., Felig P. and Conn, H. Q. (1973) Hyperglycaemia in Laennes cirrhosis. The role of portasystemic shunting. *N. Engl. J. Med.*, **290**, 239–242.
10. Benoit, J. N., Zimmenman, B., Premen, A. J., Liang, V., Go W. and Granger, D. N. (1986) Role of glucagon in splanchnic hyperemia of portal hypertension. *Am. J. Physiol.*, **251**, G674–677.
11. Geraghty, J. G., Angerson W. J. and Carter, D. C. (1989) Portal venous pressure and portasystemic shunting in experimental portal hypertension. *Am. J. Physiol.*, **257**, G52–57.
12. McLean, E. K., McLean, A. E. M. and Sutton, P. M. (1969) Instant cirrhosis, *Br. J. Exp. Pathol.*, **50**, 502–506.
13. Ishise, S., Pegram, B. L., Yamomoto, J., Kitamura Y. and Frohlich, E. D. (1980) Reference sample microsphere method: cardiac output and blood flows in consious rats. *Am. J. Physiol.*, **239**, H443–449.
14. Chojkier, M. and Groszmann, R. J. (1981) Measurement of portal-systemic shunting in the rat by using gamma-labelled micrispheres. *Am. J. Physiol.*, **240**, G371–357.
15. Ardill, J. (1979) Radioimmunoassay of G1 hormones. *Clin. Endo. Metab.*, **8**, 265–280.
16. Flanagan, R. W. J., Buchanan K. D. and Murphy, R. F. (1974) Specifity of antibodies in the radioimmunoassy of glucagon. *Diabelologia.*, **10**, 365.
17. Sikuler, E. Polio, J., Groszman R. J. and Hendler, R. (1987) Glucagon and insulin metabolism in a portal-hypertensive rat model. *Am. J. Physiol.*, **253**, G110–115.

### INVITED COMMENTARY

Several years ago, we proposed a relationship between portosystemic shunting, glucagon and the hemodynamic consequences of chronic portal hypertension. The essence of our hypothesis was that portosystemic shunting led to increased circulating levels of glucagon which began to act as a dilator of the splanchnic vasculature. As a result, the hyperdynamic splanchnic circulation developed. In the years since this initial report, several studies have examined the role of humoral factors in portal hypertensive conditions. The work of Geraghty *et al.*, joins a growing body of literature supporting the role of glucagon as a mediator of the hemodynamic derangements in chronic portal hypertension. A major finding of the present study was a correlation between portal pressure and glucagon levels in cirrhotic rats. The authors suggested that glucagon may mediate the increased portal pressure. There are two possible mechanisms by which glucagon could raise portal pressure: 1) increased portal venous inflow and 2) increased portal vascular resistance.

Previous reports from our laboratory have clearly shown that glucagon is capable of increasing portal venous inflow in chronic portal hypertension. Furthermore, we have suggested that the increased portal inflow can account for a significant portion of the rise in portal venous pressure observed in prehepatic portal hypertension. Thus it is conceivable that the correlation between glucagon and portal pressure represents active congestion of the portal system, a result of the splanchnic vasodilation. An alternative explanation for glucagon induced changes in portal pressure could be related to the known action of glucagon on the hepatic portal system. Richardson and Withrington reported a significant correlation between portal glucagon concentrations and increased portal vascular resistance in normal dogs. Superior mesenteric vascular flow increased and hepatic arterial vascular resistance was unaltered during glucagon infusion. These data lead us to suggest that glucagon may augment portal pressure in cirrhosis by selectively constricting the hepatic portal circulation. To this end, the increased portal resistance observed by Geraghty *et al.*, may have also been related to glucagon. However, neither of the aforementioned mechanisms can be directly supported by the data presented by these investigations.

In summary, Geraghty *et al.*, propose a role for glucagon as a mediator of the portal hypertension associated with cirrhosis. Data in the literature would lead one to conclude that both increased portal venous inflow and increased hepatic portal resistance are consequences of the hyperglucagonemia of cirrhosis. Future studies by these investigators will be necessary before they can advance their hypothesis.

Joseph N. Benoit, Ph.D.

Zhi-Yong Wu, M.D., Ph.D.
Department of Physiology and Biophysics
Louisiana State University Medical Center
Shreveport, LA 7113-3932

### REFERENCES

1. Benoit, J. N., Barrowman, J. A., Harper, S. L., Kvietys P. R. and Granger, D. N. (1984) Role of humoral factors in the intestinal hyperemia associated with chronic portal hypertension, *Am. J. Physiol.*, **247**, G486–G493.
2. Benoit, J. N., Zimmerman, B., Premen, A. J., Go, V. L. W. and Granger, D. N., (1986) Role of glucagon in splanchnic hyperemia of chronic portal hypertension. *Am. J. Physiol.*, **251**, G674–G677.
3. Joh, J., Granger D. N. and Benoit, J. N. (1991) Intestinal microvascular responsiveness to norepinephrine in chronic portal hypertension; *Am. J. Physiol.*, **260**, H1135–H1143.
4. Mesh, C. L., Joh, T., Korthuis, R. J., Granger G. N. and Benoit, J. N. (1991) Intestinal vascular sensitivity to vasopressin in portal hypertensive rats. *Gastroenterology.*, **100**, 916–921.
5. Kravetz, D., Arderiu, M., Bosch, J., Fuster, J., Visa, J., Casamitjana J. and Rodes, J. (1987) Hyperglucagonemia and hyperkinetic circulation after portocaval shunt in the rat. *Am. J. Physiol.*, **252**, G257–G261.
6. Benoit, J. N., Womack, W. A., Hernandez L. and Granger, D. N. (1985) "Forward" and "Backward" flow mechanisms of portal hypertension Relative contribution in the rat model of portal vein stenosis, *Gastroenterology.*, **89**, 1092–1096.
7. Richardson P. D. I. and Withrington, P. G. (1978) The effects of intraportal infusions of glucagon on the hepatic arterial and portal venous vascular beds of the dog: Inhibition of hepatic arterial vasoconstrictor responses to noradrenaline. *Pflugers Arch.*, **378**, 135–140.

